# m6AGE: A Predictor for N6-Methyladenosine Sites Identification Utilizing Sequence Characteristics and Graph Embedding-Based Geometrical Information

**DOI:** 10.3389/fgene.2021.670852

**Published:** 2021-05-27

**Authors:** Yan Wang, Rui Guo, Lan Huang, Sen Yang, Xuemei Hu, Kai He

**Affiliations:** ^1^Key Laboratory of Symbol Computation and Knowledge Engineering of Ministry of Education, and College of Computer Science and Technology, Jilin University, Changchun, China; ^2^School of Artificial Intelligence, Jilin University, Changchun, China

**Keywords:** m^6^A, machine learning, graph embedding, feature fusion, CatBoost

## Abstract

N^6^-methyladenosine (m^6^A) is one of the most prevalent RNA post-transcriptional modifications and is involved in various vital biological processes such as mRNA splicing, exporting, stability, and so on. Identifying m^6^A sites contributes to understanding the functional mechanism and biological significance of m^6^A. The existing biological experimental methods for identifying m^6^A sites are time-consuming and costly. Thus, developing a high confidence computational method is significant to explore m^6^A intrinsic characters. In this study, we propose a predictor called m6AGE which utilizes sequence-derived and graph embedding features. To the best of our knowledge, our predictor is the first to combine sequence-derived features and graph embeddings for m^6^A site prediction. Comparison results show that our proposed predictor achieved the best performance compared with other predictors on four public datasets across three species. On the *A101* dataset, our predictor outperformed 1.34% (accuracy), 0.0227 (Matthew’s correlation coefficient), 5.63% (specificity), and 0.0081 (AUC) than comparing predictors, which indicates that m6AGE is a useful tool for m^6^A site prediction. The source code of m6AGE is available at https://github.com/bokunoBike/m6AGE.

## Introduction

N^6^-methyladenosine (m^6^A) is one of the most prevalent RNA post-transcriptional modifications. It was first found in mammalian RNA in 1974 (Desrosiers et al., 1974). Subsequently, m^6^A modification was observed in various species, such as Saccharomyces cerevisiae (Schwartz et al., 2013), Arabidopsis (Luo et al., 2014), humans and mouse (Dominissini et al., 2012). Research shows that m^6^A sites are enriched in long internal exons and 3′UTRs around stop codons rather than randomly distributed in the genome (Dominissini et al., 2012; Meyer et al., 2012; Wan et al., 2015). It has been reported that m^6^A modification is associated with many biological processes, including but not limited to protein translation and localization (Meyer and Jaffrey, 2014), mRNA splicing and stability (Nilsen, 2014), RNA localization and degradation (Meyer and Jaffrey, 2014). Therefore, precisely identifying m^6^A sites contributes to understanding the regulatory mechanism and biological significance of m^6^A modification.

High-throughput techniques have enabled locating the m^6^A sites in genomes. MeRIP-Seq (or m6A-Seq), a combination of immunoprecipitation and next-generation sequencing technology, has successfully mapped m^6^A in several species genomes (Dominissini et al., 2012; Schwartz et al., 2013; Wan et al., 2015). In 2015, Chenet al. developed photo-crosslinking-assisted m^6^A-sequencing (PA-m^6^A-seq) which provided a high-resolution (about 23nt) mammalian map (Chen et al., 2015a). MeRIP-Seq and PA-m6A-seq can only locate the high methylation regions of m^6^A rather than the exact positions. In the same year, Linder produced a single-nucleotide resolution map of m^6^A sites using a new technology termed miCLIP (Linder et al., 2015). However, the current experimental methods face a lot of limitations and expensive costs. With the rapid development of computational methods, it is possible to use machine learning algorithms to predict m^6^A. Hence, building advanced models to predict m^6^A sites is significant for the following research of m^6^A.

Chen et al. (2015b) proposed the first predictor named iRNA-Methyl for m^6^A sites in Saccharomyces cerevisiae, using three physical-chemical properties of dinucleotide and SVM classifier. WHISTLE (Chen et al., 2019) integrates genomic features besides the sequence features to train a predictor with SVM classifier. Liu and Chen (2020) developed a computational method called iMRM for detecting different RNA modifications simultaneously with XGBoost classifier. Recently, deep learning methods show better performance trend in bioinformatics problems. DeepM6ASeq (Zhang and Hamada, 2018), BERMP (Huang et al., 2018), Gene2vec (Zou et al., 2019), DeepPromise (Chen et al., 2020), and im6A-TS-CNN (Liu et al., 2020) establish deep learning frameworks by using convolutional neural network (CNN) layers and gated recurrent unit (GRU) to seek the m^6^A sites on DNA/RNA sequence level on the same dataset as SRAMP (Zhou et al., 2016). In this study, seven kinds of sequence-derived features are employed to encode RNA sequences, including CTD (Tong and Liu, 2019), Pseudo k-tuple Composition (PseKNC) (Guo et al., 2014), nucleotide pair spectrum (NPS) (Zhou et al., 2016), nucleotide pair position specificity (NPPS) (Xing et al., 2017), nucleotide chemical properties and density (NCP-ND) (Golam Bari et al., 2013), electron-ion interaction pseudopotentials (EIIP) (Nair and Sreenadhan, 2006), and bi-profile Bayes (BPB) (Shao et al., 2009). Besides, graph embedding methods are innovatively introduced to distill the potential structure information. Firstly, a network is constructed by mapping each sample of the dataset to a node. Secondly, the three graph embedding methods SocDim (Tang and Liu, 2009), Node2Vec (Grover and Leskovec, 2016), and GraRep (Cao et al., 2015) are used to learn the distributed representation of the sample in an unsupervised manner. At last, all the feature vectors are merged as the input of model. The predictive results show that m6AGE improves the performance of identifying m^6^A sites.

## Materials and Methods

### Datasets

The m^6^A sites of different species share different consensus motifs. The adenosines lying within the consensus motif are considered to be the potential methylation sites. The samples in the dataset are RNA sequence segments with the potential methylation sites at their center. The samples with the m^6^A sites experimentally annotated are put into the positive dataset, whereas the other samples are put into the negative dataset.

There have been many datasets across multiple species for training m^6^A site predictors. We have collected four datasets that involve three species: Saccharomyces cerevisiae, Arabidopsis thaliana, and human. The following are details of these datasets.

*A101*. Wang extracted *A.thaliana* m^6^A sites from the m^6^A peak data of Luo et al. (2014) and Wan et al. (2015). The dataset (Wang and Yan, 2018) Wang built contains 2,518 positive samples and 2,518 negative samples. Every sample in the dataset is a 101nt RNA sequence segment.

*A25*. Luo obtained 4,317 m^6^A peaks detected both in Can-0 and Hen-16 strains. After removing the sequences with more than 60% sequence similarity, Chen et al. (2016) obtained 394 positive samples. The same number of negative samples were selected randomly from sequences without the m^6^A site. The length of every sample is 25nt.

*S21*. Chen further constructed this dataset (Chen et al., 2015c) based on the previous work (Chen et al., 2015b). They selected 832 RNA sequence segments as the positive samples in the training set whose distances to the m^6^A-seq peaks are less than 10nt. Then, 832 of 33,280 RNA sequence segments with non-methylated adenines were selected randomly as negative samples in the training set. The rest 475 RNA sequences with methylated adenine and 4750 of 33,280 RNA sequences with non-methylated adenine constitute the independent testing dataset. The length of every sample is 21nt.

*H41*. Chen obtained the m^6^A-containing sequences in *Homo sapiens* from RMBase (Chen et al., 2017). All the m^6^A sites in these sequences conform to the RRACH motif. The dataset contains 1,130 positive samples and 1,130 negative samples. The length of every sample is 41nt.

### Construction of Input Feature

**FIGURE 1:**
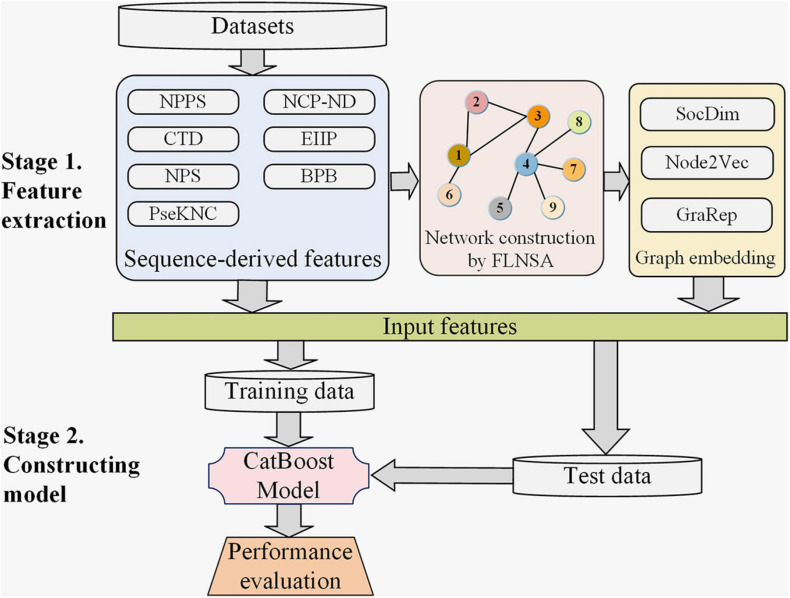
The computational framework of our predictor m6AGE. There are two main stages in the construction of m6AGE. Stage 1. Sequence-derived features are extracted, and graph embeddings are learned. Sequence-derived feature encoding methods directly encode RNA sequences into numerical vectors, including CTD, NPS, PseKNC, NPPS, NCP-ND, EIIP, and BPB feature encoding method. All the sequences are mapped to nodes of a network, and then their graph embeddings (SocDim, Node2Vec, and GraRep) are learned in an unsupervised manner. At last, the sequence features and graph embeddings are merged as input features. Stage 2. We divide the data into training data and test data with a ratio of 4:1. The training data is used to train a CatBoost model. The test data is used to evaluate the performance of our predictor.

Conventional machine learning models require numerical vectors as input features. The feature extraction methods selected have an important impact on the performance of the model. To fully characterize the context of m^6^A sites, seven sequence-derived features were used. In addition, we build a network based on the whole dataset, by mapping each sample to node and the similarity between samples to edges in the network, and then use graph embedding (neighborhood-based node embedding) methods to extract features in an unsupervised manner. The computational framework of our predictor is illustrated in Figure 1. In the following, we will introduce the sequence-derived features and the graph embeddings, respectively.

#### Sequence-Derived Features

*CTD Feature*

CTD (Tong and Liu, 2019) is one of the global sequence descriptors. The first descriptor C (nucleotide composition) describes the percentage composition of each nucleotide in the sequence. The second descriptor T (nucleotide transition) describes the frequency of four different nucleotides present in adjacent positions. The third descriptor D (nucleotide distribution) describes five relative positions of each nucleotide along the RNA sequence which are the first one, 25%, 50%, 75%, and the last one.

*PseKNC Feature*

With the successful application of the pseudo component method in peptide sequence processing, its idea has been further extended to the study of DNA and RNA sequences feature representation. The Pseudo k-tuple Composition (PseKNC) combines the local and global sequence information of RNA (Guo et al., 2014) and transforms an RNA sequence into the following vector:

(1) $${\text{D}}_{\text{PseKNC}}={\left[d_{1},d_{2},\ldots ,d_{4^{k}},d_{4^{k}+1},\ldots ,d_{4^{k}+\lambda }\right]}^{T}$$

where,

(2) $$d_{u}=\{\begin{array}{c} \frac{f_{u}}{\sum_{i=1}^{4^{k}}f_{i}+w\,\sum_{j=1}^{\lambda }\theta_{j}}\left(1\leq u\leq 4^{k}\right)\\ \frac{w\,\theta_{u-4^{k}}}{\sum_{i=1}^{4^{k}}f_{i}+w\,\sum_{j=1}^{\lambda }\theta_{j}}\left(4^{k}<u\leq 4^{k}+\lambda \right) \end{array}$$

where *d*_*u*_(*u* = 1,2,…,4^*k*^) is the occurrence frequency of the u-th k-nucleotide in this RNA sequence; the parameter *w* is the weight factor; the parameter λ is the number of totals counted tiers of the correlations along an RNA sequence. The *j*-tier correlation factor θ_*j*_ is defined as follows:

(3) $$\theta_{j}=\frac{1}{L-j-1}\,\sum_{i=1}^{L-j-1}\Theta \,\left(R_{i}\,R_{i+1},R_{i+j}\,R_{i+j+1}\right),\left(j=1,2,\ldots ,\lambda ;\lambda <L\right)$$

The correlation function Θ(,) is calculated by the following formula:

(4) $$\Theta \,\left(R_{i}\,R_{i+1},R_{i+j}\,R_{i+j+1}\right)=\frac{1}{\mu }\,\sum_{\nu =1}^{\mu }{\left[P_{\nu }\,\left(R_{i}\,R_{i+1}\right)-P_{\nu }\,\left(R_{i+j}\,R_{i+j+1}\right)\right]}^{2}$$

where μ is the number of RNA physicochemical properties used. *R_i_R*_*i+1*_ is the dinucleotide at position *i* of this RNA. *P*_ν_(*R*_*i*_*R*_*i* + 1_) is the standardized numerical value of the ν-th RNA physicochemical properties for dinucleotide *R_i_R*_*i +1*_.

Six RNA physicochemical properties are considered: “Rise”, “Roll”, “Shift”, “Slide”, “Tilt”, “Twist”.

*NPS Feature*

The nucleotide pair spectrum (NPS) (Zhou et al., 2016) encoding method describes the RNA sequence context of the site by calculating the occurrence frequency of all *k*-spaced nucleotide pairs in the sequence. The *k*-spaced nucleotide pair *n*_1_{*k*}*n*_2_ means that there are *k* arbitrary nucleotides between *n*_*1*_ and *n*_*2*_, and its occurrence frequency is calculated as follows:

(5) $$d_{n_{1}\,\left\{k\right\}\,n_{2}}=\frac{C\,\left(n_{1}\,\left\{k\right\}\,n_{2}\right)}{L-k-1}$$

where *C*(*n*_1_{*k*}*n*_2_) is the count of *n*_1_{*k*}*n*_2_ in this RNA sequence, and *L* is the sequence length. The parameter *k* ranges from 1 to *d*_*max*_. The parameter *d*_*max*_ is set to 3, so this encoding method transforms an RNA sequence into a vector **D**_*NPS*_ with a dimension of 4×4×3=48.

*NPPS Feature*

The nucleotide pair position specificity (NPPS) (Xing et al., 2017) encoding method extracts statistical information by calculating the frequency of single nucleotide and *k*-spaced nucleotide pairs at specific locations. Based on the positive training dataset, we can get the frequency matrix

(6) $$F_{s}^{+}=\left[\begin{array}{ccc} f_{s\,\left(A,1\right)}^{+} & \cdots & f_{s\,\left(A,L\right)}^{+}\\ \vdots & \ddots & \vdots \\ f_{s\,\left(G,1\right)}^{+} & \cdots & f_{s\,\left(G,L\right)}^{+} \end{array}\right]$$

(7) $$F_{d}^{+}=\left[\begin{array}{ccc} f_{d\,\left(A\,A,1\right)}^{+} & \cdots & f_{d\,\left(A\,A,L-k-1\right)}^{+}\\ \vdots & \ddots & \vdots \\ f_{d\,\left(G\,G,1\right)}^{+} & \cdots & f_{d\,\left(G\,G,L-k-1\right)}^{+} \end{array}\right]$$

where the element of $F_{s}^{+}$ is the frequency of single nucleotide appearing at each location in the positive training dataset; the element of $F_{d}^{+}$ is the frequency of *k*-spaced nucleotide pair appearing at each location in the positive training dataset; and *L* is the sequence length. The frequency matrix $F_{s}^{-}$ and $F_{d}^{-}$ are calculated similarly on the negative training dataset.

Assuming that the *i*-th nucleotide is “A” and the (*i* + *k*)-th nucleotide is “C”, $p_{i}^{+}$ is calculated through conditional probability formula and frequency matrix:

(8) $$p_{i}^{+}=\frac{f_{d\,\left(A\,C,i\right)}^{+}}{f_{s\,\left(C,i+k\right)}^{+}}$$

NPPS encoding method transforms a sequence into a vector **D**_*NPPS*_ = [*p*_*k* + 2_,…,*p*_*L*_] with a dimension of *L*−*k*−1, where $p_{i}=p_{i}^{+}-p_{i}^{-}$.

*NCP-ND Feature*

Different nucleotides have different chemical properties. According to the difference of ring structure (purine or pyrimidine), hydrogen bond (strong or weak), and functional group (amino or keto), nucleotide A, U, C, and G can be represented by (1, 1, 1), (0, 1, 0), (0, 0, 1), and (1, 0, 0), respectively (Golam Bari et al., 2013).

The nucleotide density (ND) is used to measure the relevance between the frequency and position of the *i*-th nucleotide *n_i_* in the sequence:

(9) $$d_{n_{i}}=\frac{1}{i}\,\sum_{j=1}^{L}t\,\left(n_{j}\right),t\,\left(q\right)=\{\begin{array}{c} 1,i\,f\,n_{j}=q\\ 0,o\,t\,h\,e\,r\,c\,a\,s\,e \end{array}$$

where *L* is the sequence length. Combined with the chemical properties of nucleotides, each sequence is transformed into a vector **D**_*NCP*−*ND*_ with a dimension of *L*×4.

*EIIP Feature*

This encoding method uses the electron-ion interaction pseudopotentials (EIIP) values (Nair and Sreenadhan, 2006) to represent the nucleotide in the sequence. The EIIP values of nucleotides A, T (we replace T with U), C, G are 0.1260, 0.1340, 0.0806, and 0.1335, respectively. Thus the dimension of the vector **D**_*EIIP*_ is equal to the sequence length.

*BPB Feature*

The Bi-profile Bayes (BPB) encoding method was first proposed by (Shao et al., 2009), and then has been successfully applied in other fields of bioinformatics. This method uses the occurrence frequency *f*_*i,n*_ of the *i*-th nucleotide *n* to estimate the posterior probability *p*_*i,n*_, and transforms a sequence into the following vector:

(10) $${\text{D}}_{B\,P\,B}=\left[f_{1,n}^{+},f_{1,n}^{-},f_{2,n}^{+},f_{2,n}^{-},\ldots ,f_{L,n}^{+},f_{L,n}^{-}\right]$$

where *n* is the *i*-th nucleotide of the sequence; $f_{i,n}^{+}$ denotes the frequency of nucleotide *n* appearing at the *i*-th position of the sequence in the positive training dataset, while $f_{i,n}^{-}$ denotes the frequency of nucleotide *n* appearing at the *i*-th position of sequence in the negative training dataset. *L* is the sequence length. The dimension of the vector **D**_*BPB*_ is 2×*L*.

#### Graph Embeddings

*Network Construction*

To extract the graph embedding feature of each sample, we construct a network based on the whole dataset. Each sample in the dataset is taken as a node, and the relationships between samples are taken as edges. Generally, edges exist two similar sample nodes. The fast linear neighbor similarity approach (FLNSA) (Zhang et al., 2017, 2019) is a method to extract “sample-sample” similarity, which has been successfully applied to many bioinformatics classification tasks. In this study, FLNSA is utilized to calculate the similarity between samples.

First, we extract sequence-derived features and use the feature fusion strategy to transform all the samples in the dataset into *n*-dimensional vector {*x*_1_,*x*_2_,…,*x*_*m*_}, where *x*_*i*_(0 < *i*≤*m*) is the vector of the *i*-th sample. Then these vectors are concentrated into a matrix *X* ∈ *R*^*m*×*n*^, each row of which represents a sample vector. FLNSA tries to minimize the objective function:

(11) $$\underset{w}{m\,i\,n}\frac{1}{2}\,{||X-\left(C\,⊙W\right)\,X||}_{F}^{2}+\frac{\mu }{2}\,\sum_{i=1}^{m}{||\left(C\,⊙W\right)\,\text{e}||}_{F}^{2}$$

s.t.(C⁢⊙W)⁢e=e,W≥0

where ⊙ is the Hadamard product operator; ||⋅||_*F*_ represents the Frobenius norm and μ is the regularization coefficient. **e** is an *m*-dimensional column vector with all elements equal to 1. The element *w*_*i,j*_ of matrix *W* ∈ *R*^*m*×*m*^ represents the reconstruction contribution weight of the sample *x_j_* to the sample *x_i_*, and is used to quantify the similarity between two samples. The element of indicator *C* ∈ *R*^*m*×*m*^ is

(12) $$c_{i,j}=\left\{ \begin{array}{c} 1\;x_{j}\in N\,\left(x_{i}\right)\\ 0\;x_{j}\notin N\,\left(x_{i}\right) \end{array}\right.$$

where *N*(*x*_*i*_) denotes the set of all neighbors of *x_i_*. The Euclidean distances between *x_i_* and other samples are calculated and the nearest *c*(0 < *c* < *m*) samples are selected to form *N*(*x*_*i*_). FLNSA uses the Lagrange method to get matrix *W*. After mathematical derivation, the Equation (13) is obtained.

(13) $$W_{i\,j}=\{\begin{array}{c} \frac{W_{i\,j}\,{\left(X\,X^{T}+\mu \,{\text{ee}}^{T}\right)}_{i\,j}}{{\left(\left(C\,⊙\text{W}\right)\,X\,X^{T}+\mu \,\left(C\,⊙\text{W}\right)\,{\text{ee}}^{T}\right)}_{i\,j}}\,x_{j}\in N\,\left(x_{i}\right)\\ 0\;x_{j}\notin N\,\left(x_{i}\right) \end{array}$$

Randomly generated matrix *W* was updated according to Equation (13) until convergence. Taking *W* as the adjacency matrix, an undirected weighted graph *G* is obtained. The graph embedding methods require a connected graph as input. Note that if *G* is not connected, we can increase *c* (the number of neighborhoods of a sample). Under the condition of ensuring the connectivity of the graph, the edges whose weights are lower than the threshold *t* are removed and the weights of the remaining edges are set to 1. Finally, an undirected unweighted graph is constructed based on the dataset.

*SocDim*

The social-dimension-based (SocDim) (Tang and Liu, 2009) method is proposed by Lei Tang and Huan Liu to solve the relational learning between nodes in social networks. This method extracts latent dimensions from networks and uses them as distributed representations, which involves community detection tasks.

SocDim uses Modularity (Newman, 2006) which measures community structure through degree distribution to extract potential dimensions. Modularity considers dividing the network into non-overlapping communities, measures the deviation between the network and uniform random graphs with the same degree distribution, and then obtains the modularity matrix *B* defined as follows:

(14) $$B=A-\frac{{\text{dd}}^{T}}{2\,m}$$

where *A* is the interaction matrix of the network; **d** is a column vector composed of the degrees of each node; *m* is the number of nodes. Subsequently, SocioDim extracts the dimensions from the top eigenvectors of the modularity matrix *B*.

*Node2Vec*

Node2Vec (Grover and Leskovec, 2016) attempts to design a graph embedding model that can train efficiently and retain the neighborhood information of nodes to the maximum extent. The embedding vectors of nodes are learned through the skip-gram model. Different from DeepWalk, Node2Vec proposes biased random walk instead of truncated random walk to control the search space. Node2vec considers the homophily (nodes from the same community have similar embeddings) and structural equivalence (nodes that share similar roles have similar embeddings), thus there are two classic search strategies: Breadth-first Sampling (BFS) and Depth-first Sampling (DFS).

*GraRep*

GraRep (Cao et al., 2015) proposes a graph embedding model that can be learned from weighted graphs and integrate global structure information of the graph. GraRep forms *k* different vectors by separating *k* kinds of relationships. For a specific *k*, GraRep samples a set of *k*-step paths from the graph. The *k*-step path which starts with node *v_w_* and ends with node *v_c_* is denoted as (*v*_*w*_,*v*_*c*_). For all pairs, it increases the probability of the pairs come from the graph and decreases the probability of the pairs do not come from the graph. Based on the normalized adjacency matrix, GraRep obtains *W*^*k*^ for different values of *k*, and each column vector of *W*^*k*^ represents an embedding of the node. Finally, this method concatenates all the *k*-step representations *W*^1^,*W*^2^,…,*W*^*k*^.

### CatBoost Classifier

CatBoost (Dorogush et al., 2018; Prokhorenkova et al., 2018) is an improved implementation of gradient enhanced decision trees (GDBT) algorithm developed by Yandex. It has demonstrated excellent performance on many classification and regression tasks. Compared with other advanced gradient boosting algorithms such as XGBoost (Chen and Guestrin, 2016) and lightBGM (Ke et al., 2017), CatBoost has the following advantages: (1) It can better process categorical features. (2) To solve the problem of gradient bias and prediction shift, ordered boosting is proposed instead of the classic GDBT gradient estimation algorithm. (3) The requirement of super parameter tuning is reduced.

CatBoost uses oblivious decision trees (Langley and Sage, 1994) as base predictors. As oblivious decision trees are balanced, they can prevent overfitting. Moreover, it optimizes the traditional boosting algorithm which transforms the category features into numerical features, and the algorithm of calculating the leaf value to improve the generalization ability of the model. Since the CatBoost algorithm is running on GPU, the model is trained efficiently and parallelly.

### Evaluation Metrics

Our predictor predicts whether the adenosine at the center of an RNA sequence segment is an m^6^A site. We used the following metrics to evaluate the performance of binary classification predictors: accuracy (ACC), Matthew’s correlation coefficient (MCC), sensitivity (SEN), specificity (SPE), and F1. These metrics are calculated as follows:

(15) $$A\,C\,C=\frac{T\,P+T\,N}{T\,P+F\,N+T\,N+F\,P}\times 100\%$$

(16) $$M\,C\,C=\frac{T\,P\,\text{×}\,T\,N-F\,P\,\text{×}\,F\,N}{\sqrt{\left(T\,P+F\,N\right)\,\left(T\,N+F\,P\right)\,\left(T\,P+F\,P\right)\,\left(T\,N+F\,N\right)}}$$

(17) $$S\,E\,N=\frac{T\,P}{T\,P+F\,N}\times 100\%$$

(18) $$S\,P\,E=\frac{T\,N}{T\,N+F\,P}\times 100\%$$

(19) $$F\,1=\frac{2\,T\,P}{2\,T\,P+F\,P+F\,N}$$

where *TP* is the number of true positive samples; *TN* is the number of true negative samples; *FP* is the number of false positive samples; *FN* is the number of false negative samples.

Additionally, the receiver operating characteristic (ROC) curve is also an important measurement to evaluate the performance of classifiers, and the area under receiver operating characteristic curve (AUC) is the quantitative indicator. High values of AUC indicate better performance of predictors.

## Results

We redivided the four datasets introduced in section “Datasets” into the training sets and test sets with the ratio of 4:1, respectively. The training datasets were used to train models and the test datasets were utilized to evaluate model performance. Due to the difference between datasets, we selected suitable sequence-derived features for each dataset. For *A101*, the PseKNC, CTD, and NPS features were selected; For *A25*, the EIIP, NPPS, NPS, PseKNC, and NCP-ND were selected; For *S21*, the NPPS and NCP-ND features were selected; For *H41*, the NCP-ND, PseKNC, and NPPS features were selected.

### Comparison With Existing Predictors

In this section, we compared the performance of our predictor m6AGE with several other state-of-the-art predictors, including M6A-HPCS (Zhang et al., 2016), Targetm6A(Li et al., 2016), RAM-NPPS (Xing et al., 2017), M6APred-EL (Wei et al., 2018), and DeepM6ASeq (Zhang and Hamada, 2018). M6A-HPCS uses PseDNC and DACC features and a support vector machine (SVM) classifier to identify m^6^A sites. Targetm6A utilizes position-specific kmer propensities (PSKP) feature and SVM classifier. RAM-NPPS uses the NPPS feature and SVM classifier to identify m^6^A sites. M6APred-EL creates an ensemble model with PseKNC, PSKP, and NCP-ND features. DeepM6ASeq develops a deep learning framework and uses one-hot encoding for the identification of m^6^A sites. The predictor M6A-HPCS, M6APred-EL, Targetm6A, and RAM-NPPS were reproduced faithfully, and their parameters were optimized by grid search with five-fold cross-validation. All predictors were trained and evaluated on the same dataset for fairness of comparison.

The evaluation results were summarized in Table 1. We employed ACC, MCC, SEN, SPE, and AUC as evaluation metrics, and compared the evaluation metrics of m6AGE with five other predictors on three datasets: *A101*, *A25*, and *H41*. As shown in Table 1, our predictor m6AGE achieved all optimal values on three datasets, except for SEN and SPE on the *A25* dataset, and AUC on the *H41* dataset.

**TABLE 1:** The performance of m6AGE against other existing predictors.

Datasets	Predictors	Metrics				
		ACC (%)	MCC	SEN (%)	SPE (%)	AUC
A101	m6AGE	**89.11**	**0.7822**	90.49	**87.68**	**0.9500**
	M6A-HPCS	86.43	0.7286	86.64	86.22	0.9284
	Targetm6A	87.36	0.7471	87.65	87.06	0.9358
	RAM-NPPS	83.86	0.6777	86.44	81.21	0.9077
	M6APred-EL	86.02	0.7205	85.63	86.43	0.9055
	DeepM6ASeq	87.77	0.7595	**93.32**	82.05	0.9419
A25	m6AGE	**87.97**	**0.7708**	74.65	98.85	**0.8867**
	M6A-HPCS	68.35	0.3577	61.97	73.56	0.7238
	Targetm6A	82.91	0.6542	76.06	88.51	0.8370
	RAM-NPPS	82.91	0.6538	**77.46**	87.36	0.8621
	M6APred-EL	87.34	0.7642	71.83	**100.00**	0.8464
	DeepM6ASeq	77.85	0.5515	67.61	86.21	0.8054
H41	m6AGE	**90.93**	**0.8325**	**81.94**	**100.00**	0.9181
	M6A-HPCS	71.46	0.4336	64.76	78.22	0.7765
	Targetm6A	90.49	0.8249	81.06	**100.00**	**0.9205**
	RAM-NPPS	90.49	0.8249	81.06	**100.00**	0.9051
	M6APred-EL	89.82	0.8136	79.74	**100.00**	0.9132
	DeepM6ASeq	86.50	0.7566	73.57	99.56	0.9051

*The optimal value of each evaluation metric is marked in bold.*

On the *A101* dataset, m6AGE obtained the optimal ACC, MCC, SPE, and AUC with 89.11%, 0.7822, 87.68%, and 0.9500, which is 1.34%, 0.0227, 5.63%, and 0.0081 higher than the suboptimal predictor DeepM6ASeq, respectively.

On the *A25* dataset, m6AGE obtained the optimal ACC, MCC, and AUC with 87.97%, 0.7708, and 0.8867. Its Acc and MCC is 0.63% and 0.0066 higher than the suboptimal value of predictor M6APred-EL. Its AUC is 0.0246 higher than the suboptimal value of predictor RAM-NPPS.

On the *H41* dataset, m6AGE obtained the optimal ACC, MCC, SEN, and SPE with 90.93%, 0.8325, 81.94%, and 100%, which is 0.44%, 0.0076, 0.88%, and 0 higher than the predictor Targetm6A and RAM-NPPS, respectively.

**FIGURE 2:**
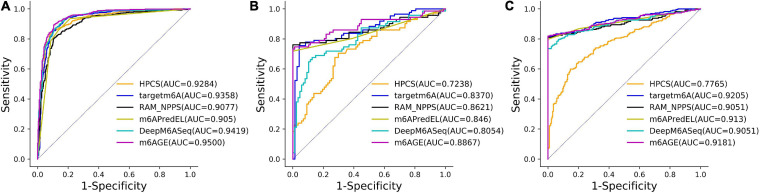
The ROC curves of m6AGE and comparing predictors on three datasets. **(A)** The ROC curves on the *A101* dataset. **(B)** The ROC curves on the *A25* dataset. **(C)** The ROC curves on the *H41* dataset.

The ROC curves of these predictors on three datasets were plotted in Figure 2. As shown in Figure 2, our predictor outperformed other predictors on the *A101* and *A25* datasets. Although the AUC of m6AGE on dataset *H41* is lower than other predictors, m6AGE achieved the optimal value of ACC, MCC, SEN, and SPE. These evaluation results demonstrate that our predictor m6AGE is superior to other predictors in terms of these three datasets.

### Performance on Imbalanced Dataset

The non-m^6^a sites on mRNA are much more than m^6^A sites, so testing the performance of our predictor on imbalanced datasets is of great importance. The imbalance ratio of the *S21* dataset is about 1:4. We redivided the *S21* dataset, and randomly selected 80% samples as the training set, and the remaining 20% samples as the test set.

CatBoost solves the imbalance data issues by setting weights for each class or sample. The weight of each class is generally inversely proportional to the number of its samples. The metrics F1 and MCC are usually used as the evaluation criteria for imbalanced datasets (Zhao et al., 2018; Wang et al., 2019; Dou et al., 2020). We compared m6AGE with five other predictors on the *S21* dataset.

The evaluation results were summarized in Table 2. The optimal value of each evaluation metric is marked in bold. As shown in Table 2, our predictor m6AGE got the optimal values of F1, MCC, and AUC with 0.5723, 0.4593, and 0.8103.

**TABLE 2:** The performance of different predictors on *S21* dataset.

Predictors	Metrics				
	SEN (%)	SPE (%)	F1	MCC	AUC
m6AGE	68.68	83.02	**0.5723**	**0.4593**	**0.8103**
HPCS	71.70	46.63	0.3622	0.1459	0.6330
Targetm6A	70.57	76.73	0.5260	0.3984	0.7818
RAM-NPPS	66.42	81.49	0.5440	0.4218	0.7778
M6APred-EL	**78.59**	75.20	0.5554	0.4433	0.7899
DeepM6ASeq	63.77	**83.38**	0.5460	0.4253	0.8056

*The optimal value of each evaluation metric is marked in bold.*

**FIGURE 3:**
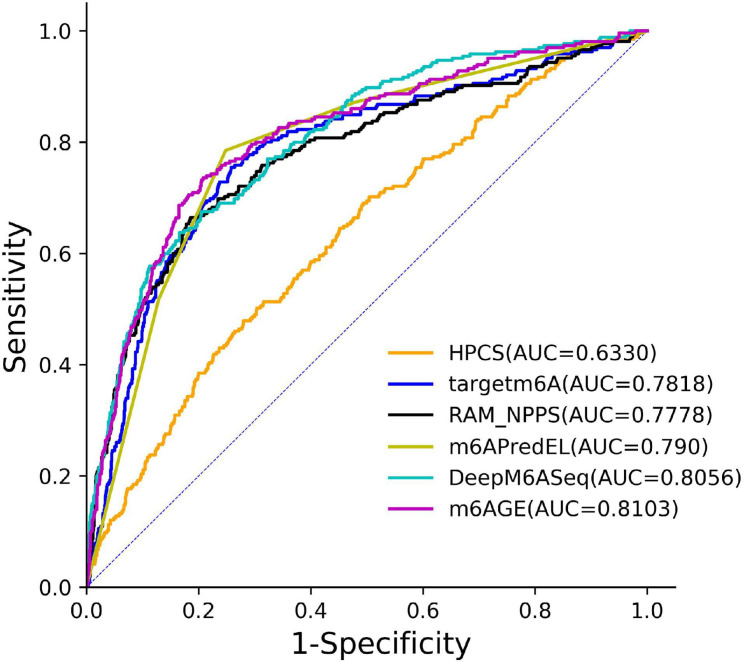
The ROC curves of m6AGE and comparing predictors on the *S21* datasets.

The ROC curves of these predictors on the *S21* dataset were plotted in Figure 3. As shown in Figure 3, our predictor outperformed other predictors on the *S21* dataset.

### Comparison With Different Classifiers

To further demonstrate the effectiveness of CatBoost, we compared it with other popular classifiers, including Random Forest, Logistic Regression, and Decision Tree, which are commonly and widely used in bioinformatics classification tasks. All classifiers were trained and assessed under the same conditions for a fair comparison.

The prediction results were summarized in Table 3. We compared the prediction results with three other classifiers on the *A101*, *A25*, and *H41* dataset. The evaluation metrics used are ACC, MCC, SEN, SPE, and AUC. As shown in Table 3, CatBoost achieved all optimal metrics on three datasets, except for SPE on the *A101* dataset and SEN on the *A25* and *H41* dataset.

**TABLE 3:** The performance of different classifiers.

Datasets	Classifiers	Metrics				
		ACC (%)	MCC	SEN (%)	SPE (%)	AUC
A101	CatBoost	**89.11**	**0.7822**	**90.49**	87.68	**0.9500**
	Random forest	87.67	0.7534	87.04	88.31	0.9377
	Logistic regression	89.00	0.7800	89.07	**88.94**	0.9489
	Decision tree	80.99	0.6197	82.39	79.54	0.8096
A25	CatBoost	**87.97**	**0.7708**	74.65	98.85	**0.8867**
	Random forest	87.34	0.7642	71.83	**100.00**	0.8729
	Logistic regression	79.11	0.5767	74.65	82.76	0.8562
	Decision tree	81.65	0.6349	**84.51**	79.31	0.8191
H41	CatBoost	**90.93**	**0.8325**	81.94	**100.00**	**0.9181**
	random forest	89.38	0.8031	79.74	99.11	0.9098
	Logistic regression	86.95	0.7422	82.38	91.56	0.9125
	Decision tree	86.28	0.7258	**85.46**	87.11	0.8629

*The optimal value of each evaluation metric is marked in bold.*

### Feature Importance Analysis

**FIGURE 4:**
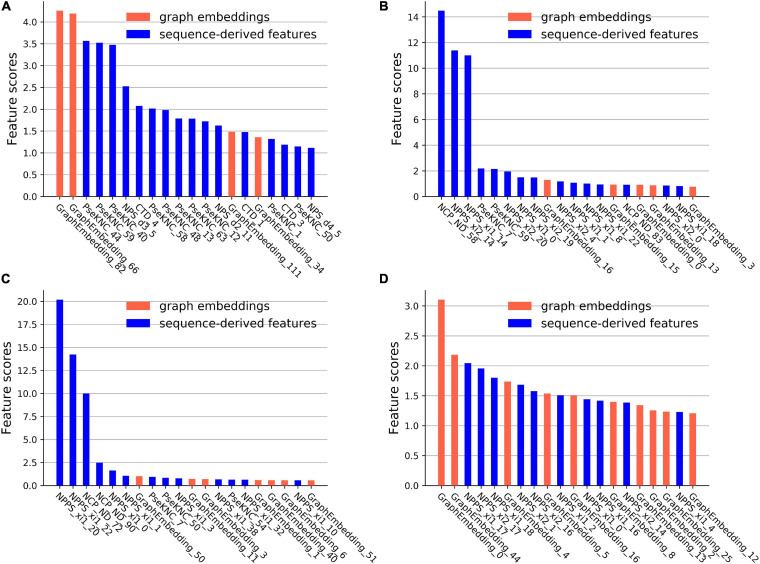
The feature importance scores on the four datasets. **(A)** The feature importance scores on the *A101* datasets. **(B)** The feature importance scores on the *A25* datasets. **(C)** The feature importance scores on the *H41* datasets. **(D)** The feature importance scores on the *S21* datasets.

CatBoost can output the scores of feature importance, which reflect the contributions of the features in specific feature space for identifying m^6^A sites. The first 20 important features and their scores on the four datasets were plotted in Figure 4.

On the *A101* dataset, the first three important sequence-derived features are “PseKNC_44”, “PseKNC_59”, and “PseKNC_40”, which correspond to the occurrence frequency of “GUA”, “UGU”, and PseKNC_40 respectively, On the *A25* dataset, the first three important sequence-derived features are “NCP_ND_58”, “NPPS_xi2_14”, and “NPPS_xi1_14,” which correspond to the position +1 (Assuming that the position of m^6^A site is 0), +2 and +4, +2 and +3, respectively; On the *H41* dataset, the first three important sequence-derived features are “NPPS_xi1_20”, “NPPS_xi1_22”, and “NCP_ND_72”, which correspond to the position 0 and +1, +2 and +3, −3, respectively; On the *S21* dataset, the first three important sequence-derived features are “NPPS_xi1_17”, “NPPS_xi2_17”, and “NPPS_xi1_18,” which correspond to the position +6 and +7, +6 and +8, +7 and +9, respectively.

In addition, graph embeddings account for 20%, 25%, 35%, and 50% of the top 20 important features in the four datasets, respectively, which indicates that graph embeddings could supplement the information of the sequence-derived features.

## Discussion

The methods for extracting sequence features are indispensable for building a reliable predictor. Contributing sequence features, such as the physical and chemical properties of nucleotides, the frequency of k-nucleotides, and the frequency of specific positions, can fully reflect the information related to the m^6^A site recognition. In this study, we integrated and selected suitable sequence-derived features for each dataset. However, most of the feature encoding methods are based on the primary sequence, and only a few of them calculate the frequency of nucleotides in the training dataset, so it is difficult to obtain more helpful information from the whole dataset. This paper innovatively introduces a feature extraction method based on the graph embedding methods as a supplement to sequence-derived features. First of all, a network is constructed based on the whole dataset and sequence-derived features. Samples are abstracted as nodes of the network, and the similarity relationships between samples are abstracted as edges. This network reflects global information of the whole dataset. Then, graph embedding (neighborhood-based node embedding) methods are used to learn the feature representation of each node in an unsupervised manner. The graph embedding features of samples contain the related information with other samples. Finally, we integrate sequence-derived features and graph embeddings based with the feature fusion strategy. Therefore, the final input features can reflect the information of samples more comprehensively.

It is also significant to choose an appropriate classifier. CatBoost is a GBDT algorithm, which shows excellent performance in many classification tasks. Because of its good effect of restraining overfitting and fast running, the CatBoost algorithm is selected to train our predictor m6AGE.

To further prove the effectiveness of our predictor, we compare the evaluation results with that of other existing m^6^A site predictors. The results show that our predictor m6AGE outperforms other existing methods. In the future, we will apply m6AGE to more m^6^A site datasets and seek more suitable graph embedding methods. It is worth mentioning that the computational framework proposed in this study is possible to extend to other bioinformatics site identification tasks.

The source code of m6AGE is available at https://github.com/bokunoBike/m6AGE. Users can download and run it on the local machines. The data is imported through the file paths of the positive training set, negative training set, and test set. Then m6AGE is trained and generates prediction results. Note that the corresponding python packages need to be installed first (see GitHub page for details). For a new dataset, our predictor will automatically select the appropriate sequence-derived features (or specified by the users in the corresponding configuration file) according to the feature importance scores.

## Conclusion

The identification of N^6^-methyladenosine (m^6^A) modification sites on RNA is of biological significance. In this study, a novel computational framework called “m6AGE” is proposed to predict and identify the m^6^A sites on mRNA. Our predictor combines sequence-derived features with the features extracted by graph embedding methods. The context information of sites is directly extracted from primary sequences by the sequence-derived features, and the global information is extracted by the graph embeddings. Experiments showed that the proposed m6AGE achieved successful prediction performance on four datasets across three species. It could be expected that m6AGE would be a powerful computational tool for predicting and identifying the m^6^A modification sites on mRNA.

## Data Availability Statement

Publicly available datasets were analyzed in this study. Codes and data are available here: https://github.com/bokunoBike/m6AGE which contains detailed steps to run m6AGE.

## Author Contributions

YW and RG conceived the algorithm and developed the program. RG, YW, and SY wrote the manuscript and prepared the datasets. YW and SY helped with manuscript editing, design. XMH, LH, and KH helped to revise the manuscript. All authors contributed to the article and approved the submitted version.

## Conflict of Interest

The authors declare that the research was conducted in the absence of any commercial or financial relationships that could be construed as a potential conflict of interest.
